# Mover Is a Homomeric Phospho-Protein Present on Synaptic Vesicles

**DOI:** 10.1371/journal.pone.0063474

**Published:** 2013-05-28

**Authors:** Saheeb Ahmed, Nina Wittenmayer, Thomas Kremer, Jan Hoeber, Asha Kiran Akula, Henning Urlaub, Markus Islinger, Joachim Kirsch, Camin Dean, Thomas Dresbach

**Affiliations:** 1 European Neuroscience Institute, Goettingen, Germany; 2 Center of Anatomy, University of Goettingen Medical School Goettingen, Germany; 3 Institute for Anatomy & Cell Biology, University of Heidelberg Medical School, Heidelberg, Germany; 4 Department of Neuroanatomy, Medical Faculty Mannheim, Heidelberg University, Heidelberg, Germany; 5 Bioanalytical Mass Spectrometry Group, Max Planck Institute for Biophysical Chemistry, Goettingen, Germany; University of Nebraska Medical Center, United States of America

## Abstract

With remarkably few exceptions, the molecules mediating synaptic vesicle exocytosis at active zones are structurally and functionally conserved between vertebrates and invertebrates. Mover was found in a yeast-2-hybrid assay using the vertebrate-specific active zone scaffolding protein bassoon as a bait. Peptides of Mover have been reported in proteomics screens for self-interacting proteins, phosphorylated proteins, and synaptic vesicle proteins, respectively. Here, we tested the predictions arising from these screens. Using flotation assays, carbonate stripping of peripheral membrane proteins, mass spectrometry, immunogold labelling of purified synaptic vesicles, and immuno-organelle isolation, we found that Mover is indeed a peripheral synaptic vesicle membrane protein. In addition, by generating an antibody against phosphorylated Mover and Western blot analysis of fractionated rat brain, we found that Mover is a *bona fide* phospho-protein. The localization of Mover to synaptic vesicles is phosphorylation dependent; treatment with a phosphatase caused Mover to dissociate from synaptic vesicles. A yeast-2-hybrid screen, co-immunoprecipitation and cell-based optical assays of homomerization revealed that Mover undergoes homophilic interaction, and regions within both the N- and C- terminus of the protein are required for this interaction. Deleting a region required for homomeric interaction abolished presynaptic targeting of recombinant Mover in cultured neurons. Together, these data prove that Mover is associated with synaptic vesicles, and implicate phosphorylation and multimerization in targeting of Mover to synaptic vesicles and presynaptic sites.

## Introduction

Neurotransmitter release at fast chemical synapses relies on sets of evolutionarily conserved proteins that mediate the regulated exocytosis, retrieval and re-use of transmitter containing synaptic vesicles (SVs). With remarkably few exceptions, the molecules mediating SV exocytosis at active zones are structurally and functionally conserved between vertebrates and invertebrates with nervous systems, such as Drosophila and *C. elegans*, and some are even conserved between vertebrates and yeast. Consistent with such highly conserved roles, ablation or perturbation of the function of these molecules leads to severely impaired synaptic transmission. These studies have revealed the role of several conserved proteins in distinct steps in the exocytotic pathway. The trans-SNARE complex formed by the plasma membrane proteins syntaxin and SNAP-25, and the SV protein synaptobrevin-2/ vamp-2 mediates the exocytotic fusion reaction [1]. Synaptotagmins confer calcium sensitivity to the fusion reaction, thus allowing for tight excitation-secretion coupling by influx of calcium into the nerve terminal [2]. Munc-13 is important for the tight tethering of SVs to the plasma membrane and appears to confine SV exocytosis to active zones, where secretion exclusively occurs. Moreover, Munc-13 confers fusion competence onto SVs. Thus, Munc-13 generates a pool of docked and fusion competent SVs at the active zone [3], [4]. In addition, proteins of the Sec1/Munc18 (SM) family are crucial for SV exocytosis [5], presumably by controlling SNARE-complex topology and function [6], [7] .

Interestingly, in addition to the above-mentioned conserved proteins, a few presynaptic proteins occur only in vertebrates, including synuclein, a SV-associated protein, and bassoon, a large scaffolding protein of the presynaptic active zone. These proteins may confer vertebrate-specific functions to synaptic transmission. Bassoon is important for the replenishment of SVs at active zones in inner hair cells [8] and at hippocampal mossy fiber synapses [9]. In cultured neurons prepared from bassoon mutant mice, a subset of presynaptic boutons fails to recycle SVs [10] and knockdown of bassoon in cultures prepared from knockout mice lacking the related active zone scaffolding protein piccolo leads to a reduction in the number of SVs at synaptic junctions [11]. Thus, these proteins appear to have more subtle effects than those that are evolutionarily conserved. Apart from synuclein, only one vertebrate-specific protein, Mover (also called TPRGL1 and SVAP30), has been hypothesized to be present on SVs. Mover was found in a yeast-2-hybrid assay using bassoon as a bait, reasoning that some of the interaction partners of such a scaffolding protein might themselves be vertebrate-specific. We named the 266 amino acid prey protein Mover because it was localized to mossy-fibre nerve terminals in the hippocampus, and was indeed vertebrate-specific [12]. Peptides of Mover have been found in three different screens, including approaches to identify self-interacting proteins [13], phosphorylated proteins of synaptosomes [14], and SV proteins, where the predicted protein was termed SVAP30 [15]. The corresponding mouse gene was also called TPRGL1 [16]. Here, we tested the predictions arising from these screens and found that Mover is indeed a homomeric phospho-protein associated with SVs.

## Materials and Methods

### Ethics statement

All research involving animals was done in accordance with the guidelines of the Goettingen and Heidelberg University animal welfare committees and German animal welfare laws.

### Antibodies and mammalian expression constructs

Antibodies: Mover; described in [12]; phospho-Mover (described below); tubulin (Sigma, DM1A); myc-tag (Santa Cruz, clone 9E10); flag-tag (Sigma, clone M2); Erc2/CAST (Synaptic Systems, cat. 143–003); rab3a (Synaptic Systems, clone 42.2); synaptobrevin-2 (Synaptic Systems, clone 69.1); synapsin-1 (Synaptic Systems, clone 46.1); synaptophysin (Sigma, SVP-38, for immunofluorescence) and synaptophysin G95 provided by R. Jahn [17]; GFP (Abcam, cat. GFP6556, for immunofluorescence); GFP (Synaptic Systems, cat. 132–002); Rab-GDI (Synaptic System, cat. 130–001); LDH (Chemicon, SC-33781).

Rhodamine-phalloidin was purchased from Sigma. For mammalian expression, Mover-GFP was generated by subcloning the full-length Mover cDNA into pEGFP-N1 (Clontech) with EGFP carrying the A207K mutation. GFP-Mover, and the deletion constructs GFP-Mover 1–90, 1–180, 91–266, 91–180, and 181–266 were generated by subcloning Mover downstream of EGFP in the pEGFP-C1 expression vector (Clontech). Mover-myc and myc-Mover were subcloned into the pCMV promoter construct (Stratagene). The palm-Mover-flag was constructed by subcloning the palmitoylation consensus site of GAP-43 (MLCCMRRTKQVEKNDEDQKI) upstream of Mover and a flag tag downstream of Mover in the pCMV vector.

### Subcellular fractionation of adult rat brain

For subcellular fractionation, Sprague-Dawley rats were sacrificed in accordance with the German guidelines for the humane care and use of laboratory animals. Fractionation was performed as previously described [18]. Rat brains were homogenized in homogenization buffer (320 mM sucrose, 4 mM HEPES-KOH, pH 7.4 in a glass-teflon or Potter-Elvehjem homogenizer (10 strokes at 900 rpm). The resulting homogenate (H) was centrifuged for 10 min at 1000 g to remove cell debris and pellet nuclei (P1). Supernatants (S1) were pooled and centrifuged for 15 min at 10,000 g to obtain a crude synaptosomal fraction (P2), and crude brain cytosol (S2).

Synaptosomes were osmotically lysed by adding 9 vol of ice-cold ddH_2_O containing protease-inhibitors (Roche) and homogenized in a glass-teflon homogenizer (3 strokes at 2000 rpm). The lysed synaptosomes were centrifuged for 20 min at 25,000 g at 4°C to spin down lysed synaptosomal membranes (LP1). The SV containing supernatant (LS1), containing SVs and synaptosomal cytosol, was further ultracentrifuged for 2 h at 200,000 g to separate SVs (LP2) from the synaptic cytosolic fraction (LS2). The LP2 fraction was resuspended in 40 mM sucrose and subjected to a continuous sucrose gradient centrifugation (from 0.05 M sucrose to 0.8 M sucrose) for 4 h at 82,500 g and 4°C. To obtain the synaptic plasma membrane fraction, the 0.8 M–1.2 M sucrose interface was collected, diluted to 0.32 M sucrose with 5 mM HEPES-NaOH; pH 7.4 and centrifuged for 20 min at 32,000 g at 4°C to pellet synaptic plasma membranes (SPM) and separate them from the supernatant containing synaptosomal cytosol (SCyt).SVs from the gradient interface were further purified using size-exclusion chromatography on a CPG-column (using controlled pore glass beads) [19], which separates large membrane structures (Peak1) from SVs.

### Membrane flotation assay

The LP2 fraction was resuspended in 300 µl gradient buffer (20 mM HEPES, pH 7.4, 150 mM NaCl, 1 mM dithiothreitol) including 55% sucrose with or without 1% Triton X-100 and homogenized using a 25-gauge needle. Samples were incubated for 30 min on ice and then centrifuged for 10 min at 4°C and 2400 g to remove air bubbles which would hinder placement of the suspension under the gradient. The suspension was layered under a 25–52.5 % sucrose gradient using a syringe and centrifuged at 100,000 g for 16 h in a SW50 rotor (Beckman). 300 µl fractions were collected and analysed by Western blotting and immunodetection. In this assay membranes and membrane-bound proteins float up into the gradient. Upon solubilization all proteins, except for those present in lipid rafts, are extracted from membranes and therefore remain at the bottom of the gradient [20].

### Mass Spectrometry of purified synaptic vesicle fractions

Vesicle proteins were separated by 1D SDS-PAGE [21]. After Coomassie blue staining, all visible bands were excised, cut into approximately 1 mm^2^ pieces, and subjected to in-gel trypsinization [22]. The extracted peptides were analyzed by liquid chromatography-tandem mass spectrometry (LC-MS/ MS) on a Q-TOF Ultima mass spectrometer (Waters), and proteins were identified in the NCBI nonredundant database, using Mascot (Matrix Science, London) as a search engine. To determine the false-discovery rate [23], the data (PKL files) were searched against a randomized NCBInr database.

### Immunogold labeling of synaptic vesicles

For immunogold labeling, previously established methods were followed [24]. Briefly, purified SVs were applied to perforated plastic grids and fixed with 2 % paraformaldehyde and 0.2 % glutaraldehyde for 10 min, incubated in TBS containing 0.02 % glycine and 0.5% BSA for 10 min. Anti-synaptophysin G95 [17] antibody was then added to this solution and samples were incubated for 1 h at room temp. The grids were then washed two times with 0.5 % BSA/TBS solution and incubated with 0.5 % BSA/TBS solution containing secondary antibody-conjugated gold particles for 30 min at room temp. Grids were then washed four times with 0.5 % BSA/TBS solution and stained with uranyl acetate and recorded with a Philips CM 120 electron microscope at a magnification of 27,500x.

### Immuno-isolation of synaptic vesicles

Affinity-purified rabbit antiserum directed against GST-tagged Mover, or mouse monoclonal antibody directed against synaptophysin (Sigma) were coupled to Protein A magnetic beads (µMACS Protein A microbeads, Miltenyi Biotec GmbH) in 1 mM PBS-EDTA for 1 h at 4°C. Antibody-coated beads were then added to whole brain LS1 fractions in the presence of 1 mM EDTA, 1 mM EGTA and protease inhibitors (Roche). Magnetic beads were separated from the immunodepleted supernatant and washed five times with 1 mM PBS-EDTA. Bound vesicles were eluted in Laemmli buffer. Eluates and immunodepleted supernatants were separated by 12.5% SDS-PAGE and analysed by Western blot using antibodies directed against Mover, synaptophysin, Rab-GDI and LDH.

### Carbonate stripping of synaptic vesicles

Purified SVs from a CPG column [19] were incubated with 100 mM Na_2_CO_3_ pH 11.1 or sucrose buffer (320 mM sucrose, 4 mM HEPES-KOH; pH 7.4) for 30 min on ice, as previously described [25]. Following incubation vesicles were centrifuged at 88,700 g for 1 h and the pellet was resuspended in SDS buffer. Equal volumes of resuspended samples were subjected to SDS-PAGE and Western blotting analysis.

### Glutamate release from synaptosomes

For the assay of glutamate release from synaptosomes, purified synaptosome suspensions were stirred for 15 min at 37°C. Subsequently, 1.3 mM CaCl_2_ or 0.5 mM EGTA was added, with glutamate dehydrogenase (Sigma type II, 34 U) and 1 mM NADP, and solutions were incubated for 4 min. A final concentration of 50 mM KCl was then added as indicated. Generation of NADPH was monitored by absorbance at 360 nm [26].

### Production and validation of the phospho-Mover antibody

An antiserum in rabbits was generated against the peptide sequence RDTVDSAGpTSPTAVL, which includes a predicted phosphorylation site at threonine 13 of the sequence RDTVDSAGpTSPTAVLAAGEDAGAGRPGAGTPLR.Q [14]. A crude IgG-fraction was then prepared by differential ammonium sulfate precipitation. To validate the specificity of the phospho-Mover antibody, parallel immunoblots with Mover and phospho-Mover antibodies were performed on LP2 fractions. Dephosphorylated controls were generated by treating 30 µg of LP2 with 200 U of Lambda-protein phosphatase (NEB) in a reaction buffer consisting of 1 mM MnCl, 50 mM HEPES, 100 mM NaCl, 2 mM DTT, 0.01% Brij 35; pH 7.5 for 30 min at 30°C. The same reaction supplemented with 200 mM of the phosphatase inhibitor sodium orthovanadate (Sigma) served as a control for the dephosphorylation reaction. After 30 min the reaction was stopped by the addition of Laemmli buffer, samples were boiled at 95 °C and immediately subjected to gel electrophoresis.

### Dephosphorylation of Mover

CPG column purified SVs were incubated with 200 units of Lambda Protein Phosphatase (New England BioLabs) in a reaction buffer consisting of 1 mM MnCl, 50 mM HEPES, 100 mM NaCl, 2 mM DTT and 0.01 % Brij 35 pH 7.5 for 30 min at 30°C. After incubation samples were centrifuged at 88,700 g for 1 h. The pellets and supernatants were analyzed by SDS-PAGE and Western blot analysis.

### Synaptic vesicle association of Mover in stimulated synaptosomes

Synaptosomal preparations were incubated for 10 min at 37°C in Krebs-Henseleit (KH) buffer (125 mM NaCl, 5 mM KCl, 2.7 mM CaCl_2_, 1.3 mM MgSO_4_, 10 mM glucose, 25 mM HEPES/Tris; pH 7.4), or KH buffer containing 1 mM EGTA, 1 µM okadaic acid to phosphorylate proteins, or high (45 mM) KCl. Subsequently, each synaptosomal preparation was fractionated to obtain a crude SV fraction as described above. Equal volumes of the crude SV fractions from each condition were subjected to Western blot analysis and tested for differences in Mover or synapsin protein levels.

### Yeast-2-Hybrid assay

Screening was performed using the L40 yeast strain harboring HIS3 and Beta-galactosidase as a reporter gene. Nucleotide sequences encoding the entire open reading frame of rat Mover were subcloned into the lexA fusion vector pHyblexZeo (Invitrogen) and used to screen an adult mouse brain cDNA library constructed in the pPC86 vector containing the GAL4 activation domain (Invitrogen). Approximately 2×10^7^ clones of a mouse cDNA library were screened. Positive clones from the initial screen were isolated, sequenced and re-transformed to validate their ability to bind to the Mover construct. Mover deletion constructs were subsequently cloned into the pPC86 vector in an attempt to further define the binding site required for Mover homomerization.

### Co-immunoprecipitation

For co-immunoprecipitation analysis, transfected HEK293 cells were harvested in IP-Lysis buffer (50 mM Tris-HCl pH 7.5; 150 mM NaCl; 2 mM EDTA; 0.5%; NP40; Complete protease inhibitor (Roche). After centrifugation at 15.000 g for 10 min cell lysates were preincubated with 10 μl of Protein A/G sepharose beads for 1 h at 4°C to reduce unspecific binding. After removal of the beads the lysates were incubated with monoclonal anti-myc antibodies (Santa Cruz) for 1 h at 4°C. 30 μl of a 50 % slurry of Protein A/G were added, and the mixture was incubated on a shaker over night at 4°C. The beads were pelleted at 5.000 g for 30 s and washed three times with IP-lysis buffer. Bound proteins were eluted by incubation for 10 minutes at 95°C in 4× SDS sample buffer. The samples were analysed by SDS-PAGE and Western blotting.

### Cell-based homomeric interaction assays

Vero cells were cultured in DMEM (Gibco) supplemented with 10% FBS (Pan Biotech) and L-Glutamine, on glass coverslips coated with 500 µl 0.04% Polyethyleneimine. Cells were transfected at 60–80% confluence using the calcium-phosphate method. For cells growing in 24-well plates, 0.5–2 µg of plasmid DNA in 18 µl dH_2_O was mixed with 2 µl of 2.5 M CaCl_2_, followed by the addition of 20 µl transfection buffer at pH 7.01 or pH 7.05. This mix was incubated at room temperature for 20 min after which 40 µl was added to each well. After 24 hours, health and transfection efficiency of transfected cells was assessed by light and fluorescence microscopy, respectively. Cells were then fixed in 4% paraformaldehyde for 20 min, washed twice with PBS and incubated for 45 min in 500 µl blocking buffer (10% FBS, 5% sucrose, 2% Albumin, 0.3% Triton X-100 in 1× PBS). Cells were then incubated with primary antibody directed against the tags (i.e. against myc, GFP or flag) in blocking buffer for 60 min at room temp or overnight at 4°C. Cells were washed three times with 1x PBS, then incubated with secondary antibody (Alexa 488 for GFP and myc, Alexa 546 for flag) in blocking buffer without FBS for 60 in at room temp. After one wash with 1x PBS, cells were stained with DAPI (Applichem) for 5 min, washed twice with 1x PBS, once in water, and then mounted in 8 µl Mowiol 4–88 mounting medium (Calbiochem). Images were acquired on a Zeiss AxioObserver inverted fluorescence microscope with an EC Plan-Neofluar M27 100×/1.30 NA oil objective and a CoolSNAP HQ^2^ CCD camera (Photometrics). Merged images of multi-channel images were generated using MetaMorph Offline Version 7.7.0.0 (Molecular Devices, Inc.). Fluorescence intensity profiles were determined using the “Linescan“ tool of MetaMorph. A 60 pixels long (representing 5 µm in the sample) and 10 pixel wide bar was placed on an image (the exact bar position is indicated in the figure panels). The average fluorescence intensity of the 10 pixel bar width was plotted against the position along the 60 pixel length. One line scan was performed per cell.

### Hippocampal neuron cultures and transfection

Hippocampi were dissected from E19 rats as previously described [27]. Hippocampi were treated with trypsin for 20 min at 37°C, triturated to dissociate cells, plated at 25,000–50,000 cells/cm^2^ on poly-lysine coated coverslips (Carolina Biologicals), and cultured in Neurobasal supplemented with 2% B-27 and 2 mM Glutamax (Gibco/ Invitrogen). Neurons growing on 12 mm coverslips in 24-well plates were transfected with calcium phosphate at 3–4 DIV as described previously [28]. Prior to transfection, medium was removed, saved, and replaced with 500 µl 37°C Optimem (Life Technologies) and incubated for 30–60 min. 105 µl transfection buffer (274 mM NaCl, 10 mM KCl, 1.4 mM Na_2_HPO_4_, 15 mM glucose, 42 mM HEPES, pH 7.06) was added dropwise to a solution containing 7 µg of DNA and 250 mM CaCl_2_, with gentle vortexing. This mixture was incubated for 20 min at room temp, 30 µl was added per well, and neurons incubated for 60–90 min. This medium was then removed, cells were washed 3x in 37°C Neurobasal medium, and saved medium added back to the transfected cells. Cultures were fixed on DIV 14 using 4% paraformaldehyde. Immunostaining, mounting, fluorescence microscopy and image aquisitions were performed as described above for Vero cells. Fluorescence patterns were qualified as “punctate“ when a 20× image contained at least two 50×50 µm regions outside the soma that displayed more than 5 puncta, i.e. circular or elliptic areas at least 0.5 µm along one axis with a localized fluorescence intensity that was at least threefold stronger than the adjacent areas.

## Results

**Figure 1:**
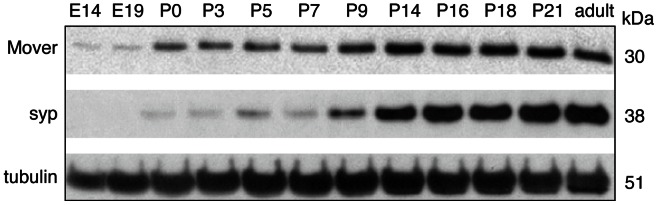
Developmental expression of Mover protein in brain. Rat brain homogenates from the indicated developmental stages between embryonic day 14 (E14) and adult were separated by molecular weight via SDS-PAGE and analyzed by Western blot using affinity-purified Mover antibodies. Tubulin and synaptophysin served as controls. N = 2 experiments.

To determine if Mover is expressed at the right time to contribute to SV composition we analyzed its developmental expression by Western blotting. Mover was detected at all developmental stages tested, from E14 through adult (Fig. 1). Its expression levels increased between E19 and P0 and remained constant after P14, similar to the SV marker synaptophysin. However, Mover was detected at earlier stages, compared to synaptophysin. In addition, Mover levels increased more gradually than those of synaptophysin. These developmental expression data are consistent with a role of Mover at synapses and during early brain development.

### Mover is a peripheral membrane protein associated with SVs

**Figure 2:**
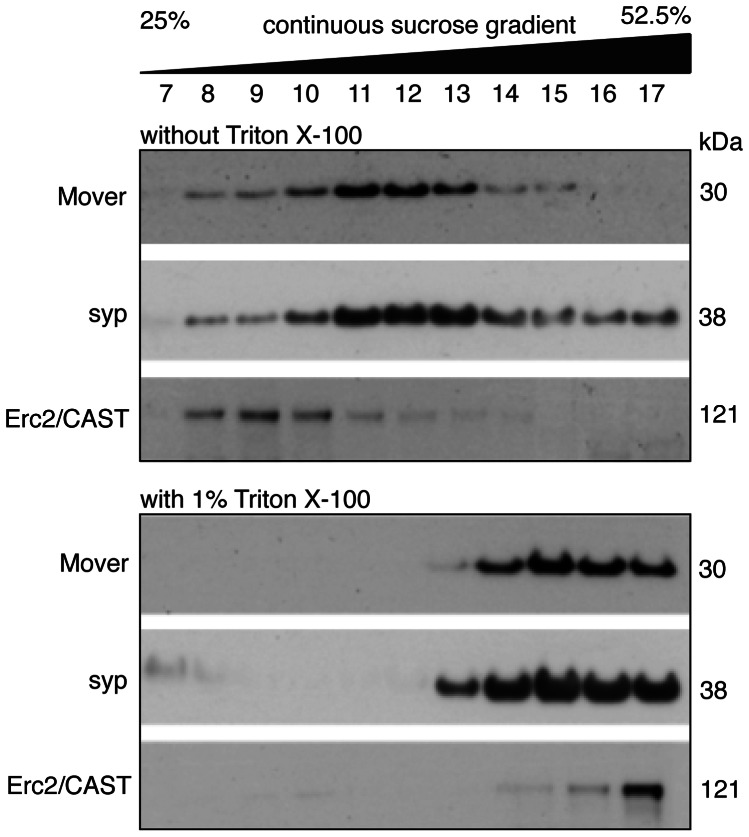
Mover associates with synaptic vesicle fractions in a membrane floatation assay. SV fractions (LP2) with and without the addition of 1% Triton X-100 to solubilize membranes were overlaid with a 25–52.5% continuous sucrose gradient and centrifuged. Without detergent, proteins associated with membranes float upwards in the gradient. With the addition of 1% Triton, only proteins attached to detergent-resistant raft-like SV membranes, such as synaptophysin, float into the gradient. Floating of Mover when membranes are intact, and co-floating of Mover with synaptophysin in 1% Triton X-100 treated SV membranes, indicate that Mover is a membrane-associated protein. N = 3 experiments.

Mover has no predicted transmembrane domain, but was detected in a proteomics screen for SV proteins [15] and in crude SV fractions [12]. This suggests that Mover is a peripheral membrane protein associated with SVs. To test this we performed several assays. We first tested whether Mover is a membrane-associated protein or a soluble protein, using a flotation assay in which membrane-associated proteins placed at the bottom of a gradient float up into the gradient, whereas purely proteinaceous complexes and soluble proteins remain at the bottom of the gradient [20]. A crude SV fraction (LP2) was layered under a continuous sucrose gradient and centrifuged. Fractions were collected from the top of the gradient to the bottom and analyzed by Western blotting for Mover, the SV-specific transmembrane protein synaptophysin, and the presynaptic cytomatrix protein ERC2/ CAST (Fig. 2). In parallel, an aliquot of the crude SV fraction was solubilized with Triton X-100 before centrifugation. Following solubilization, Mover, synaptophysin and ERC2/ CAST remained at the point of injection into the gradient (peak at fraction 15), as expected for membrane proteins. By contrast, without solubilization, i.e. with membranes intact, Mover, synaptophysin and ERC2/ CAST floated into the gradient. Mover and synaptophysin displayed a similar distribution within these fractions (Fig. 2). This demonstrates that Mover is indeed associated with membranes. Moreover, the co-distribution of Mover and synaptophysin in the same fractions is consistent with Mover being a peripheral membrane protein of SVs.

**Figure 3:**
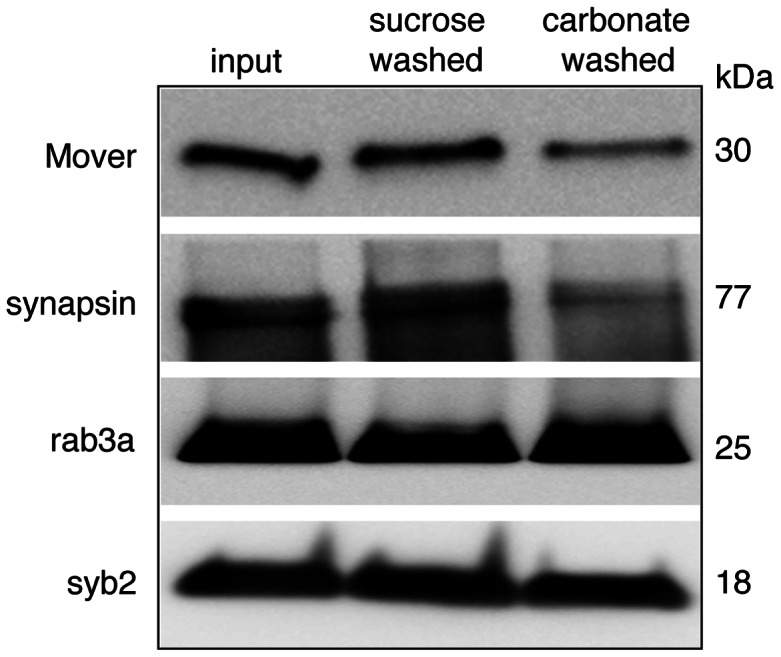
Mover is peripherally associated with synaptic vesicle membranes. Carbonate stripping by treatment of SVs with 100 mM sodium carbonate was used to determine if Mover is integrally or peripherally associated with SV membranes. Synapsin, a peripheral synaptic-vesicle membrane protein served as a positive control and was partially removed from SVs by carbonate treatment, while synaptobrevin2 (syb2), an integral SV protein, was not. Rab3a is membrane-associated but is resistant to carbonate stripping [30]. Mover was partially removed from SV membranes by carbonate stripping, at similar levels as synapsin, indicating that it is a peripheral SV membrane associated protein. N = 2 experiments.

To further corroborate this finding, we used the carbonate-stripping method [25], whereby peripherally associated membrane proteins can be stripped from SVs with sodium carbonate buffer. Purified SVs obtained using a CPG column [19] were subjected to the carbonate stripping protocol and analyzed by Western blotting (Fig. 3). Synapsin, a peripherally-associated synaptic-vesicle protein, was stripped from SVs by carbonate treatment, while synaptobrevin2 (syb2), an integral SV protein, was not. Rab3a, which is attached to the SV membrane by a polyisoprene anchor [29], was resistant to carbonate stripping, consistent with previous reports [30]. We found that Mover was stripped from SV membranes by carbonate at similar levels as synapsin, indicating that it is indeed a peripheral membrane protein, consistent with the predicted absence of a transmembrane domain.

**Figure 4:**
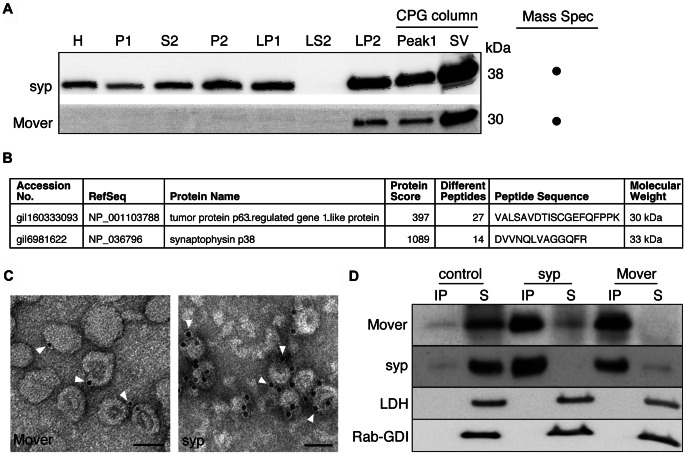
Mover is present on synaptic vesicles. (A) Mover is enriched in CPG-purified SV fractions; synaptophysin (syp), which is thought to be present exclusively on SVs, serves as a positive control (n = 2 experiments). (B) Mover, along with the positive control synaptophysin, is found in CPG-purified SV fractions analyzed by mass spectrometry (N = 1 experiment). (C) Immunogold labeling of Mover (n = 4 vesicle preparations) and synaptophysin (n = 3 vesicle preparations) in CPG-purified SV fractions imaged by electron microscopy. Scale bars are 50 nm. (D) LS1 fractions (supernatant following hypotonic lysis of synaptosomes) subjected to immunoisolation with antibodies against Mover or synaptophysin. Antibodies conjugated to Protein A beads are indicated above each lane; antibodies used for Western blotting are indicated on the left. The majority of synaptophysin-containing organelles (SVs) are immunoisolated with anti-Mover conjugated beads, and vice versa. The soluble LS1 components lactate dehydrogenas (LDH) and Rab-GDP-dissociation inhibitor (Rab-GDI) are only detected in the supernatant. Control beads without conjugated antibodies bind only trace amounts of SVs (n = 3 experiments).

To verify that Mover associates with SV membranes, we performed subcellular fractionation including purification of SVs using a CPG column, and found that Mover is enriched in the highly purified SV fraction (Fig. 4A). This pure SV fraction was subjected to mass spectrometry, where both Mover and synaptophysin were identified (Fig. 4B). Immunogold electron microscopy analysis of the same fraction using antisera against synaptophysin or Mover produced robust labelling of SVs, further indicating that Mover is a SV protein (Fig. 4C). 16.4 % percent of the SVs were positive for Mover (n = 4 vesicle preparations; 8120 SVs counted, 1332 SVs positive). 98% of the SVs were positive for synaptophysin (n = 3 vesicle preparations; 5390 SVs counted, 5282 SVs positive). 0.8% of the SVs were positive when only the secondary antibody was applied (n = 2 vesicle preparations; 1938 SVs counted, 16 SVs positive; data not shown). To further corroborate that Mover is associated with SVs, we immunoisolated SVs from hypotonically lysed synaptosomes (LS1 fraction) using magnetic beads coated with an antibody against synaptophysin. This fraction contains SVs and synaptosomal cytosol. Mover was present in the affinity-purified SV fraction, whereas the amount in the supernatant was negligible, suggesting that Mover contained in LS1 fractions is virtually completely bound to SVs (Fig. 4D). We performed the converse experiment using magnetic beads coated with an antisera against Mover. Mover beads pulled down virtually all of the synaptophysin-containing SVs in the LS1 fraction. In contrast, the soluble LS1-proteins lactate dehydrogenase and Rab-GDP-dissociation inhibitor were not immunoprecipitated by either antisera, indicating that Mover is unlikely to be a soluble contaminant in the affinity purification pellet. Together, these data strongly suggest that the vast majority of Mover present in LS1 and LP2 fractions is associated with SVs.

### Mover is a phospho-protein

**Figure 5:**
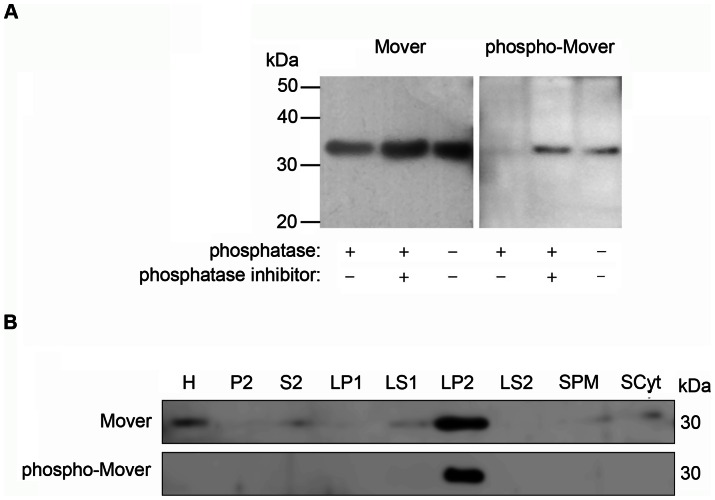
Mover is present in phosphorylated and non-phosphorylated forms on synaptic vesicles. (A) Crude synaptosomal fractions were treated with the lambda-protein phosphatase, with and without the addition of the phosphatase inhibitor sodium orthovanadate. In control conditions and in the presence of the phosphatase inhibitor, the anti-phospho-Mover antibody detects a protein band with identical molecular weight to that of non-phosphorylated Mover. This phospho-Mover protein band is reduced in intensity following phosphatase treatment (n = 3 experiments). (B) Phosphorylated Mover, similar to total Mover, is predominantly found in the SV fraction LP2. Indicated fractions are homogenate (H), crude synaptosomal fraction (P2) and corresponding supernatant (S2), synaptosomal fraction (LP1) and corresponding supernatant (LS1), SVs (LP2) and supernatant (LS2), synaptosomal membranes (SPM) and synaptosomal cytosol (Scyt). N = 2 experiments.

According to a large-scale screen for phosphorylated peptides, Mover is phosphorylated at threonine 13 [14]. To characterize the subcellular localization of phosphorylated Mover, we raised an antibody against a peptide containing amino acids 5 through 19 of rat Mover with threonine 13 phosphorylated. As shown in Figure 5A, the phospho-T13-Mover antibody detected a band of 35 kDa in rat LP2 fractions that is also recognized by the antibody against full-length Mover. Following lambda-phosphatase treatment the phospho-T13-Mover antibody failed to detect a band, whereas the full-length Mover antibody still detected Mover. Inhibition of the lambda-phosphatase with sodium orthovanadate resulted in detection of a band by the phospho-T13-Mover antibody, verifying the efficacy of phosphatase treatment. These data indicate that the antibody indeed selectively detects Mover phosphorylated at T13.

**Figure 6:**
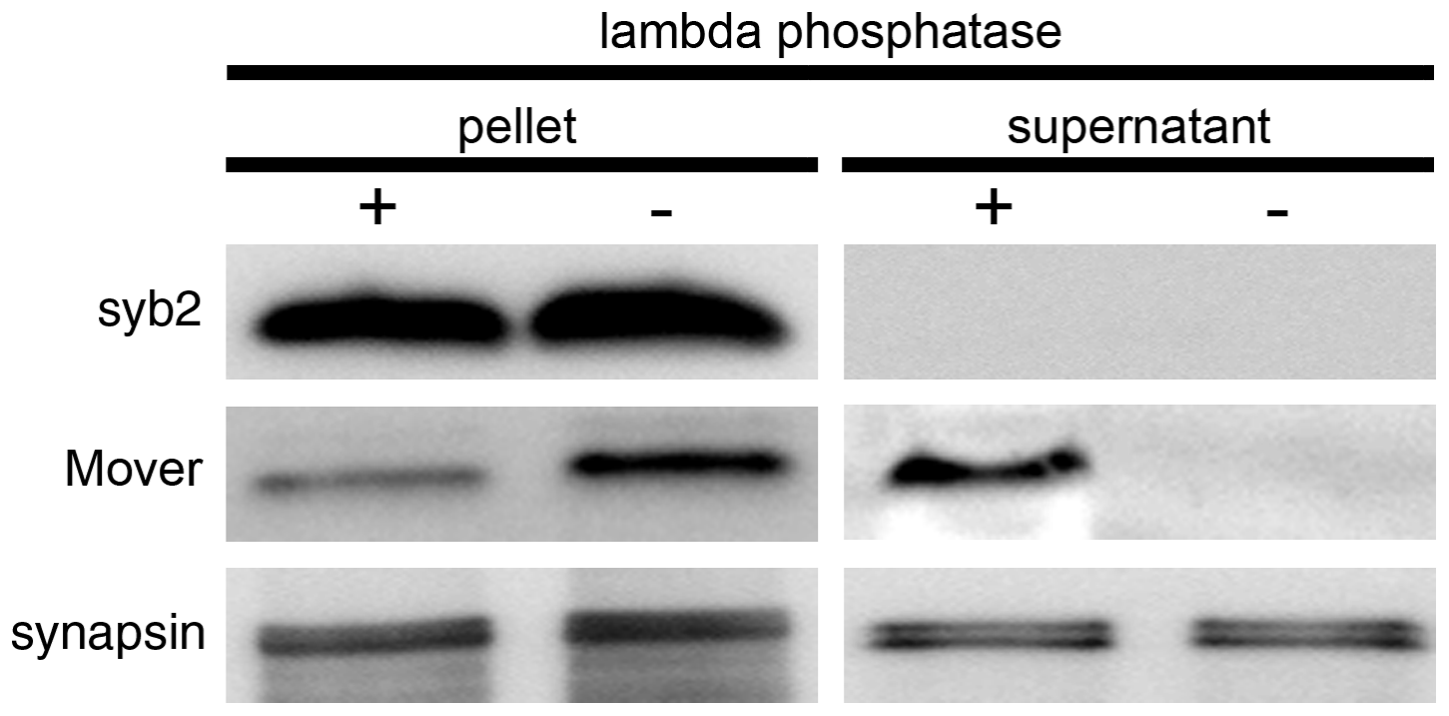
Dephosphorylated Mover dissociates from synaptic vesicles. To analyze a potential influence of phosphorylation on the localization of Mover to SV membranes, SVs were treated with lambda-protein phosphatase and then analyzed for protein content by Western blot. Dephosphorylation caused a shift of Mover immunoreactivity from the pellet to the supernatant, while the immunoreactivity for synaptobrevin and synapsin was unchanged (N = 2 experiments).

**Figure 7:**
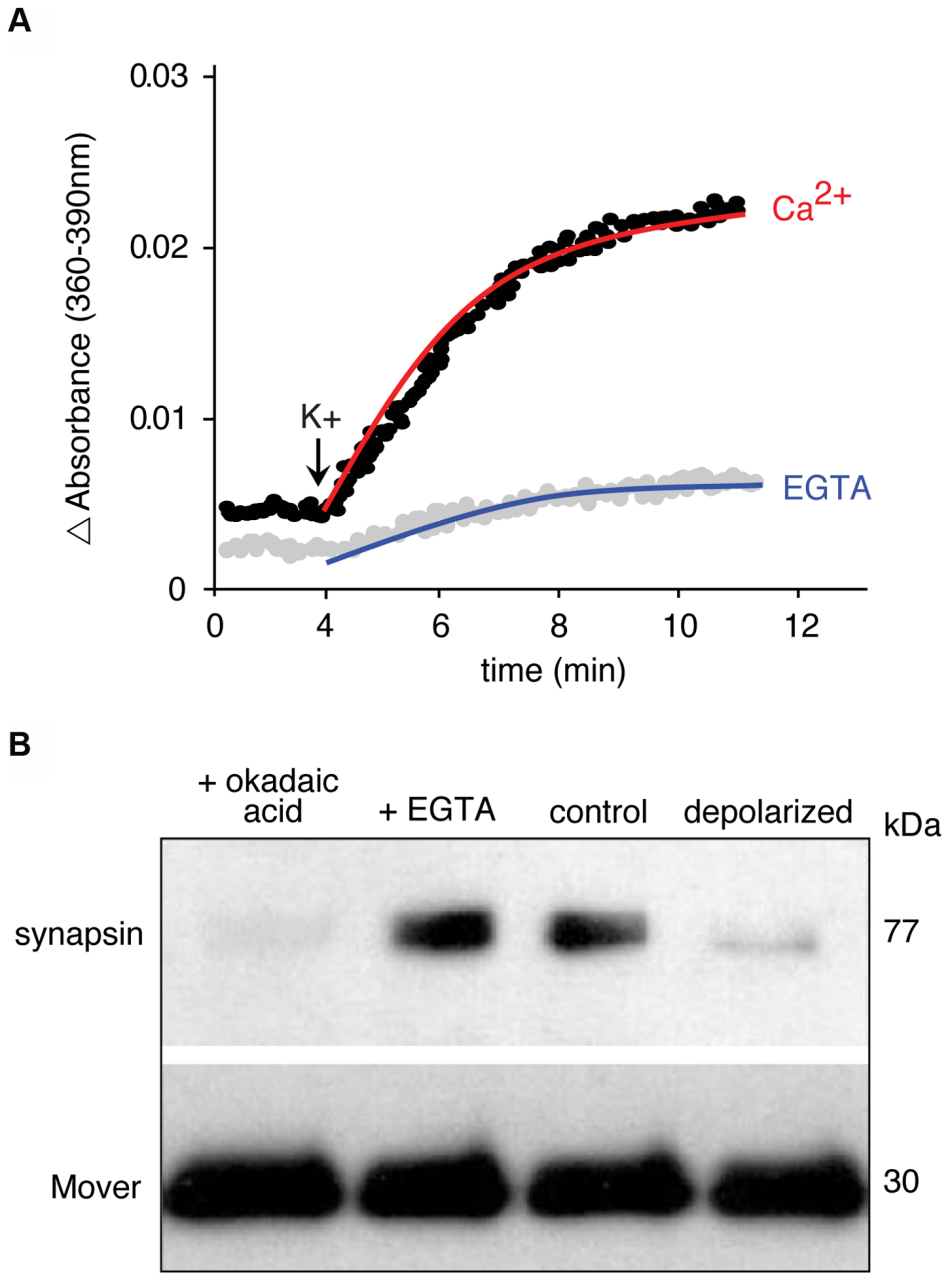
Mover does not dissociate from synaptic vesicles in response to depolarization. (A) Assay of glutamate release from synaptosomes using the fluorescence-based NADPH assay [26] to verify that SVs in synaptosomes undergo calcium-dependent fusion and exocytosis to release glutamate. Depolarization with 50 mM KCl induces glutamate release in the presence of CaCl2, but not of EGTA. (B) Synaptosomal preparations were incubated for 10 min at 37°C in control conditions, in the presence of 1 mM EGTA to chelate calcium, in 1 µM okadaic acid to phosphorylate proteins, or in depolarizing conditions. Following treatment, each synaptosomal fraction was further fractionated to obtain a crude SV fraction. Equal volumes of the crude SV fractions were then subjected to Western blot analysis to test for Mover and synapsin protein levels associated with vesicles. Mover did not dissociate from vesicles in response to depolarization, whereas synapsin did. N = 2 experiments.

Phosphorylation controls the SV association of synapsins [31]. Mover may similarly associate with SV membranes in a phosphorylation-dependent manner. To test this idea, brain fractions were analyzed using antisera against either full-length Mover or phospho-Mover. As shown in Figure 5B, both antibodies detected Mover primarily in the SV fraction (LP2), where Mover was strongly enriched. This suggests that Mover bound to SVs is phosphorylated at T13. To test whether phosphorylation is required for membrane association of Mover, we incubated CPG-purified SVs with lambda-phosphatase, centrifuged, and probed the supernatant and the SV-containing pellet for the presence of Mover. Interestingly, under de-phosphorylating conditions Mover, unlike synapsin, dissociated from SVs and appeared in the supernatant (Fig. 6). Conversely, Mover remained associated with SVs following treatment with the phosphatase inhibitor okadaic acid, while synapsin dissociated from SVs (Fig. 7). Phosphorylation and dissociation of synapsin from SVs are induced by depolarization [31]. To test whether depolarization changes the association of Mover with SVs, we depolarized synaptosomes with a high kalemic buffer (Fig. 7). We verified that the synaptosomes were functionally intact by monitoring depolarization induced, calcium dependent release of glutamate (Fig. 7A). As expected, synapsin was released into the cytosol upon depolarization. In contrast, Mover remained vesicle associated (Fig. 7B). These data suggest that Mover and synapsin are differentially regulated by phosphorylation.

### Mover undergoes homophilic interaction

**Figure 8:**
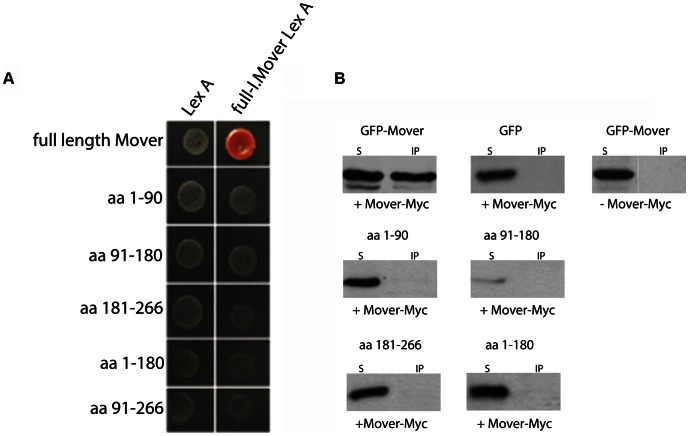
Mover is a homomeric protein. (A) Yeast 2-hybrid assay using full length Mover (full-l.) as bait. A plasmid containing only the LexA interaction domain acted as a control. Beta-galactosidase expression was only induced if full-length Mover was used as both prey (rows) and bait (columns). All deletion constructs used as prey exhibited no interaction with full-length Mover. (B) Immunoprecipitation of GFP-Mover with Mover-myc co-expressed in HEK293 cells. Sepharose-coupled antibodies against the myc epitope were used to pull down protein complexes. As in the yeast 2-hybrid assay, only full-length GFP-Mover was pulled down from the HEK cell extracts, whereas GFP and GFP-Mover deletion constructs were not. N = 2 experiments.

In a yeast-2-hybrid screen, using Mover as bait, we identified a total of 72 prey clones, each representing Mover, indicating that Mover has a strong tendency to form homomers. To localize the domain responsible for this homomeric interaction, we fused deletion constructs of Mover to the GAL4 activation domain and tested the prey clones for binding to the LexA fused full-length Mover as bait (Fig. 8A). The LexA-DNA-binding domain alone acted as a negative control (Fig. 8A, left column). Only full-length Mover constructs, but none of the Mover deletion constructs, bound to full-length-Mover bait.

To confirm these findings, n-terminally tagged GFP-Mover constructs and a c-terminally tagged Mover-myc construct were co-expressed in HEK293 cells. Following cell lysis anti-myc antibodies were used to immunoprecipitate complexes of Mover-myc and GFP-Mover. The presence of GFP-Mover was monitored by immunoblotting using a GFP antibody. As shown in Figure 8B, Mover-myc immunoprecipitated GFP-Mover, but not GFP alone. Deletion constructs that failed to interact in the yeast-2-hybrid assay also failed to co-immunoprecipitate (Figure 8B).

**Figure 9:**
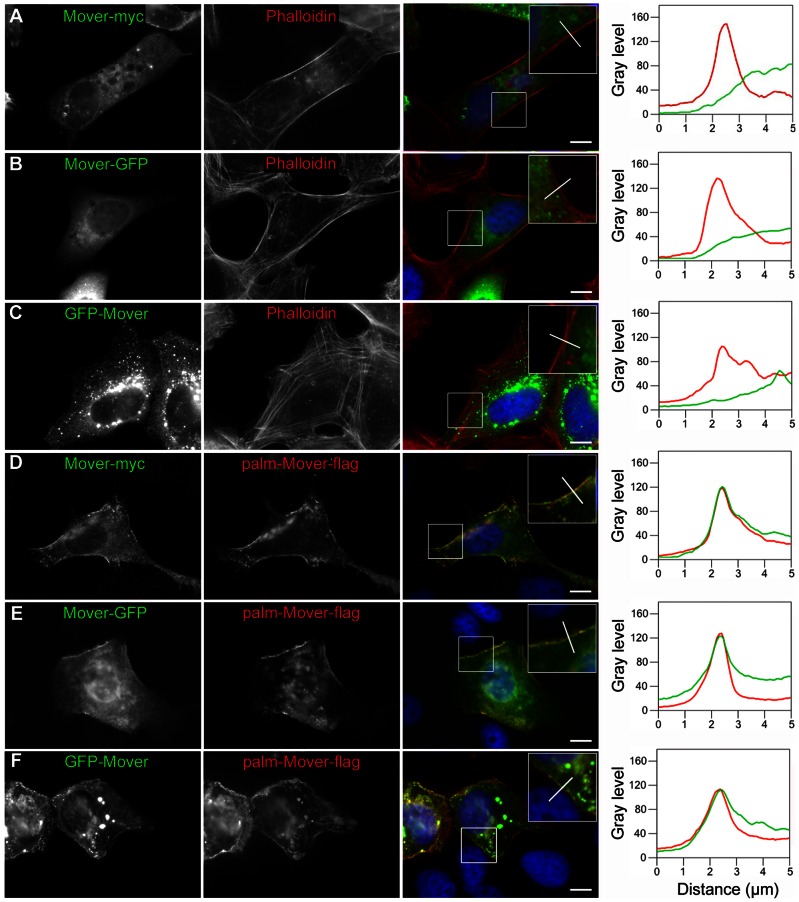
Homomeric interaction of full length Mover in Vero cells. (A–C) Mover-myc, Mover-GFP and GFP-Mover, each representing full-length versions of Mover, are either diffusely distributed or – in the case of GFP-Mover - as uniformly distributed aggregates, presumably due to some degree of homomerization. Rhodamine-Phalloidin, which stains F-actin associated with cytoplasmic actin-bundles as well as F-actin associated with the sub-plasmalemmal cell cortex, was used to delineate the cell periphery (red color in the merged images and the graphs). (D–F) Upon co-expression with the palmitoylated construct palm-Mover-flag, the constructs are recruited to the plasma membrane. Constructs were immunostained using antibodies against the tags, i.e. myc and GFP (green color in the merged images and the graphs) and flag (red color in the merged images and the graphs). The merged images also show DAPI staining in blue. For line scan analysis (right panels) a bar-shaped region of interest was placed in the image as shown (white bar, representing 5 µm×0.83 µm), and the average fluorescence occurring along its length was plotted in the graph, where 0 µm denotes the end of the bar placed in the extracellular area. Diffusely distributed constructs were characterized by a gradual decline of fluorecence from the cell interior towards the cell periphery (A–C), recruited constructs were characterized by a peak of fluorescence in the cell periphery (D–F). Scale bars are 10 µm. Zooms represent twofold magnification. N = 3 independent cultures.

**Figure 10:**
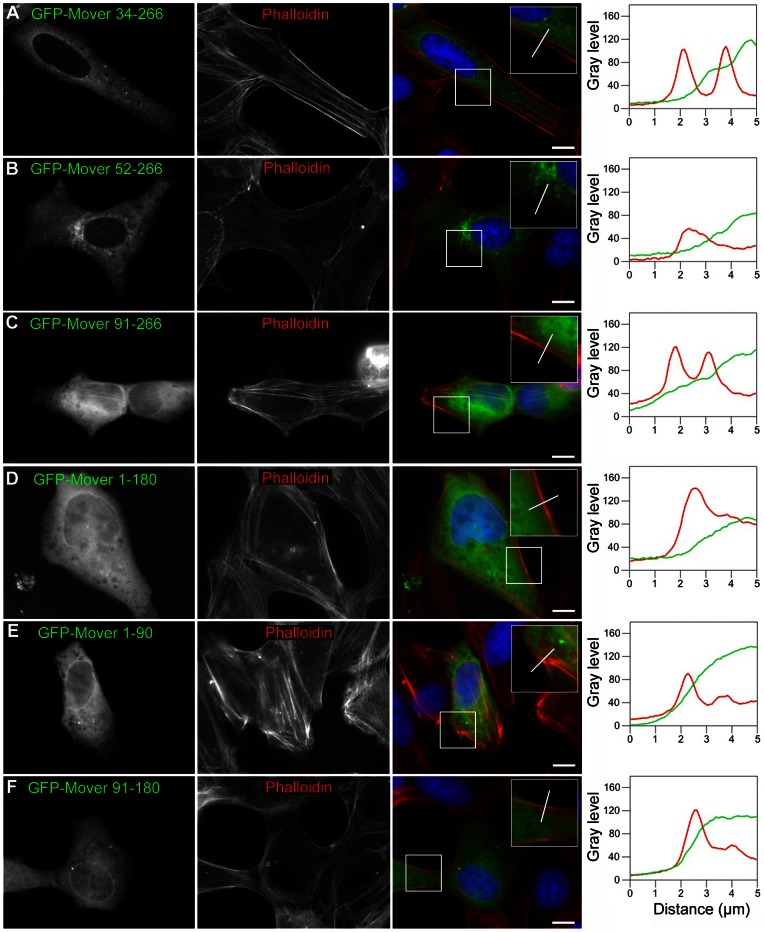
Localization of Mover deletion constructs in Vero cells. Upon expression in Vero cells, all variants of GFP-Mover carrying a deletion were diffusely distributed. Immunostaining, Phalloidin staining and line scan analysis were performed as in Figure 9. The line scan fluorescence profiles indicate that all constructs were characterized by a gradual decline of fluorescence towards the cell periphery. Scale bars are 10 µm. Boxes represent twofold magnification. N = 3 independent cultures.

**Figure 11:**
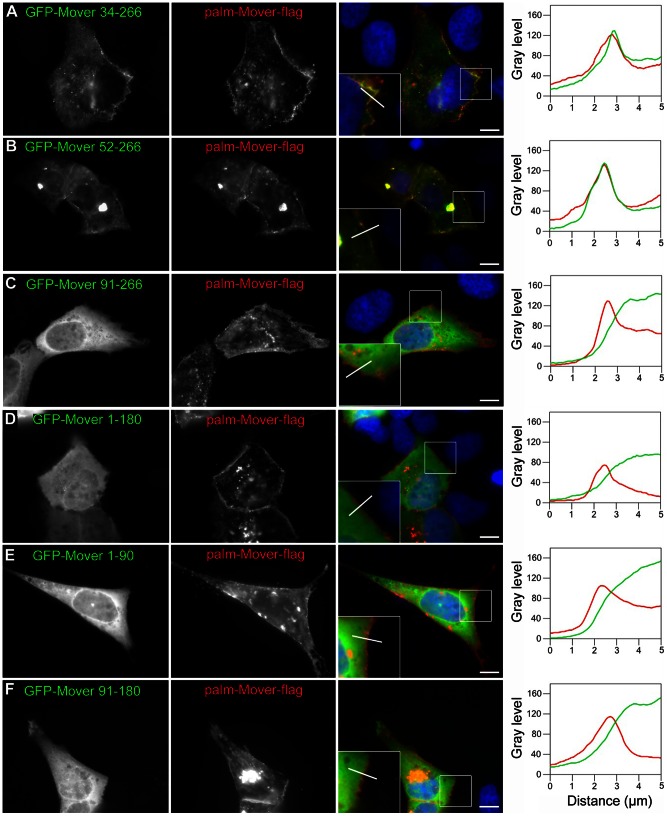
Localization of Mover deletion constructs in Vero cells expressing membrane targeted Mover. (A,B) Co-expression of GFP-Mover 34–266 or GFP-Mover 52–266 together with palm-Mover-flag recruited the GFP-constructs to the cell membrane. (C–F) Constructs with further truncations were not recruited to the membrane by palm-Mover-flag and thus are deficient in homomeric interaction. Immunostaining and line scan analysis were performed as in Figure 9. The line scan fluorescence profiles show that the fluoresence intensities for GFP-Mover 34–366 and GFP-Mover 52–266, but not of any other construct, co-peak with palm-Mover-flag immunofluorescence in the cell periphery. Scale bars are 10 µm. Boxes represent twofold magnification. N = 3 independent cultures.

To further characterize this homomeric interaction, we employed a fluorescence assay using the Vero epithelial cell line (Figs. 9–11). We chose Vero cells because of their relatively large cytoplasmic area compared to HEK293 cells, allowing for a better distinction of cytoplasmic versus plasma membrane localization of proteins by microscopy. Our goal was to test whether a plasma membrane targeted Mover construct, through homomeric interactions, would co-recruit other Mover constructs to the cell periphery. Mover-myc, Mover-GFP (with a c-terminal GFP), or GFP-Mover (with an n-terminal GFP) expressed alone were homogeneously distributed in the cytoplasm (Fig. 9A–C). We note that GFP-Mover formed aggregates, presumably due to some degree of homomerization, and these aggregates were more abundant in the cell interior than in the periphery (Fig. 9C). We used Phalloidin, which stains F-actin located in cytoplasmic bundles and in the cortical actin cytoskeleton underlying the plasmamembrane, to delineate the shape of the cells. Line scans of fluorescence intensity levels revealed a gradual decline of fluorescence intensity towards the cell periphery for Mover-myc, Mover-GFP and GFP-Mover, while Phalloidin fluorescence peaked in the cell periphery (Fig. 9A–C, right panel). In contrast, co-expression of these constructs with a plasma membrane targeted Mover construct, palm-Mover-flag, containing an n-terminal palmitoylation sequence and a c-terminal flag tag, resulted in recruitment of these constructs to the plasma membrane (9D–F). Line scans revealed that in the co-expression condition the fluorescence levels of each Mover-construct, rather than gradually declining towards the cell periphery like in 9A–C, co-peaked with the fluorescence of palm-Mover-flag in the cell periphery. To get a more quantitative estimate of the recruitment efficacy, we determined which percentage of co-transfected cells showed co-peaking of any full-length Mover construct with palm-Mover-flag. We counted cells as “positive for recruitment“ when the fluorescence profiles of palm-Mover-flag and the co-expressed myc- or GFP-tagged Mover construct co-peaked in the cell periphery. 94 percent of Vero cells showed recruitment of Mover-myc (16 of 17 cells analyzed; n = 3 independent cultures), 61 percent showed recruitment of Mover-GFP (11 of 18 cells; n = 3 independent cultures) and 94 percent showed recruitment of GFP-Mover (18 of 19 cell; n = 3 independent cultures). These data indicate that all three full-length Mover constructs are capable of interacting with palm-Mover-flag in the recruitment assay, although the c-terminal GFP-tag seems to reduce the interaction efficacy.

We then tested various Mover deletion constructs for their ability to form homomers. All of the deletion constructs were homogeneously distributed in transfected Vero cells in the absence of palm-Mover-flag, and line scan analysis revealed a gradual decline of fluorescence towards the cell periphery (Fig. 10; 21–25 cells analyzed for each construct; n = 3 independent cultures). Upon co-expression with palm-Mover-flag, GFP-Mover constructs with the first 33 or the first 52 amino acids deleted maintained the ability to form homomers, and line scans revealed co-peaking with palm-Mover flag fluorescence (Fig. 11 A,B). 71 percent of Vero cells showed recruitment of GFP-Mover 34–266 (12 of 17 cells; n = 3 independent cultures), and 86 percent showed recruitment of GFP-Mover 52–266 (18 of 21 cells; n = 3 independent cultures) based on line scan analysis. Deletion of the first 90 amino acids, however, completely abolished homomeric interaction: GFP-Mover 91–266 was not recruited by palm-Mover-flag (Fig. 11 C). Quantitative analysis revealed that 0 of 19 cells showed recruitment of GFP-Mover 91–266 (n = 3 independent cultures). A region between amino acids 52 and 91 is therefore necessary for homomeric interaction of Mover. In addition, c-terminal regions were required: GFP-Mover 1-180 and GFP-Mover 1–90 were not recruited to the membrane by palm-Mover-flag (Figs. 11D, E; 0 of 17 cells and 0 of 20 cells showed recruitment, respectively; n = 3 independent cultures). Consistent with these results, a deletion construct lacking both n- and c-terminal regions (GFP-Mover 91–180) was deficient in homomeric interaction (Fig. 11F; 0 of 15 cells showed recruitment; n = 3 independent cultures). These data support the notion that both n- and c-terminal sequences of Mover are required for homomeric interaction, and identify a region spanning amino acids 52 through 91 as a crucial component of the n-terminal sequence.

**Figure 12:**
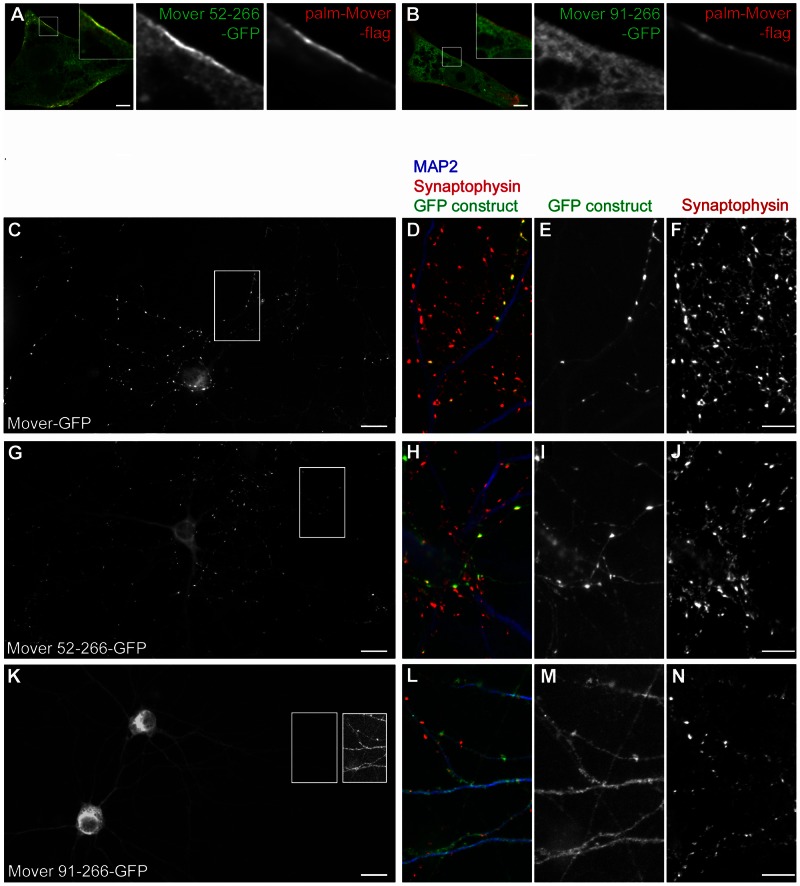
A Mover mutant deficient in homomeric interaction does not target to presynaptic sites. (A,B) Representative images showing recruitment of Mover 52–266-GFP, but not of Mover 91–266-GFP, to the plasma membrane in Vero cells expressing palm-Mover-flag. Scale bars are 5 µm, small zooms represent threefold magnification, large zooms represent sixfold magnification. (C–N) Expression of Mover-GFP, Mover 52–266-GFP and Mover 91–266-GFP in DIV 14 cultured hippocampal neurons. Mover-GFP and Mover 52–266-GFP produced a punctate fluorescence pattern, while Mover 91–266 was diffusely distributed in transfected cells (C,G,K). Note that these images were taken using a 20x objective and displayed using identical brightness settings. The diffusely labelled axons of Mover 91–266-GFP expressing cells were barely visible using these settings. The right box in K shows a brightness-enhanced and mirror-imaged copy of the left box. The boxes in C,G,K indicate areas imaged using a 40x objective and displayed at higher magnification in D–F, H–J and L–N. Immunostaining revealed extensive colocalization of punctate Mover-GFP and punctate Mover 52–266-GFP fluorescence with the synaptic vesicle marker synaptophysin (D–F and H–J). In contrast, Mover 91–266-GFP (L–N) was diffusely distributed. Axonal fluorescence was not enriched at axon-dendrite crossings (axons identified as MAP2-negative, dendrites as MAP2-positive processes. L and M show three axon-dendrite crossings). Scale bars are 20 µm in C, G, K and 10 µm in D, E, F, H, I, J, L, M and N. N = 3 independent cultures.

To test if homomeric interaction is important for the localization of Mover to SVs, we sought to compare the presynaptic targeting behavior of GFP-Mover 52–266, which does undergo homomeric interaction, with that of GFP-Mover 91–266, which does not. Upon expression in cultured hippocampal neurons GFP-Mover 52–266 produced a punctate staining pattern characteristic of presynaptic targeting, while GFP-Mover 91–266 was diffusely distributed throughout the neurons in all neurons tested (>30 neurons; n = 3; data not shown). However, full-length GFP-Mover strongly aggregated when expressed in cultured neurons, making it impossible to use as a wild-type control for presynaptic targeting. We therefore decided to use the c-terminally tagged Mover-GFP as a wild-type control, as this construct was able to undergo homomeric interaction albeit at a reduced efficacy (see quantification for Figure 9). In addition, we generated two new deletion constructs, Mover 52–266-GFP and Mover 91–266-GFP, with c-terminal GFP-tags, to match the design of Mover-GFP. We first tested these c-terminally tagged deletion constructs in the Vero cell recruitment assay. As expected, recruitment to palm-Mover-flag was observed for Mover 52–266-GFP, but not for Mover 91–266-GFP (Fig. 12A,B). We then tested these constructs for presynaptic targeting in hippocampal neurons. At 14 days in vitro, both Mover-GFP and Mover 52-266-GFP invariably produced a punctate staining pattern characteristic of presynaptic targeting (Fig. 12 C, G; 42 cells analyzed for each construct; n = 3 independent cultures). These puncta may represent Mover associated with transport entities, SV clusters or non-synaptic sites. Immunostaining revealed extensive colocalization of puncta for both constructs with the SV-marker synaptophysin (Fig. 12 D–F, H–J). To assess quantitatively whether both constructs were capable of presynaptic targeting we selected puncta contacting dendrites identified by staining for the dendritic marker MAP2 and tested the extent of their colocalization with synaptophysin. 91%+/−3.7% of Mover-GFP puncta and 96%+/−3.0% of Mover 52–266-GFP puncta were positive for synaptophysin (10 dendrites analyzed for each construct, n = 3 independent cultures). In striking contrast, Mover 91–266 was diffusely distributed in all cells (Fig. 12 C; 45 cells analyzed, n = 3 independent cultures). Immunostaining for MAP2 revealed that the construct was present in both axons (i.e. MAP2 negative processes) and dendrites. In particular, the construct did not accumulate in axons at sites of axo-dendritic contact. Fig 12 L-N). Thus, deleting a region of Mover that mediates homomeric interaction abolishes presynaptic targeting.

## Discussion

In this study we tested the hypotheses put forward by three recent proteomics screens, that Mover, a vertebrate-specific protein present at synapses, is 1) present on SVs, 2) phosphorylated, and 3) self-interacts to form homomers. Flotation and carbonate stripping experiments revealed that Mover is a peripheral membrane protein. To obtain a highly purified SV fraction from synaptosomes, we used a CPG column, which allows for the isolation of a homogeneous population of 40 nm vesicles in which 95% of the organelles are SVs [19]. Mover was confirmed to be present on these highly purified SVs by mass spectrometry, and by immunogold labeling. These results are in agreement with earlier studies in which Mover was found by mass spectrometry of samples obtained from crude SVs subjected to immunoisolation using the SV protein SV2 [15]. In our own immuno-organelle isolation experiments, anti-synaptophysin immunodepleted Mover almost entirely from the supernatant, and anti-Mover almost entirely depleted synaptophysin. This suggests that Mover and synaptophysin reside on the same organelles, i.e. SVs. Together these data strongly suggest that Mover is a *bona fide* SV protein. To determine if Mover exists in a phosphorylated form, we generated an antibody against Mover phosphorylated at threonin 13 (T13). We found that Mover is indeed phosphorylated at this site and virtually all T13-phosphorylated-Mover is on SVs. This is consistent with Mover T13 phosphorylation discovered in a screen for synaptosomal proteins that are phosphorylated during activity [14]. In a yeast 2-hybrid screen, co-immunoprecipitation experiments, and optical assays of homomerization, we found that Mover undergoes homophilic interaction, consistent with another screen for self-interacting proteins, in which Mover was found [13]. Thus, we proved, using a number of different assays, that Mover is indeed a phosphoprotein associated with SVs and that Mover undergoes homomeric interaction. In addition, our data revealed four novel features of Mover. First, Mover is expressed as early as E14, well before synaptophysin, which has a steep onset of expression at P0. Second, phosphatase treatment causes Mover to dissociate from SVs. Third, Mover remains associated with SVs in response to depolarization. Fourth, a 39-amino acid region of Mover is required for both homomeric interaction and targeting to SVs.

Our data reveal that Mover shares certain similarities with synapsin, one of the most abundant SV proteins: both are peripheral membrane proteins that are phosphorylated and undergo homomeric interaction. Dimerization of synapsins has been proposed to mediate SV clustering [32], raising the possibility that Mover may act in a similar manner. In addition to these similarities we found striking differences between these two proteins. First, synapsin dissociates from SVs upon depolarization [33]–[36]. Mover, on the other hand, does not dissociate from SVs in response to depolarization, and thus likely remains attached to SVs throughout their life cycle. Second, phosphorylation causes synapsin to dissociate from SVs [37], but dephosphorylation causes Mover to dissociate from SVs. Our fractionation data (Figures 4 and 5) indicate that virtually all Mover is associated with SVs, suggesting that Mover predominantly exists in the phosphorylated form. Thus, despite striking similarities, Mover appears to behave differently in response to depolarization and phosphorylation compared to synapsin.

Mover dissociates from SVs upon phosphatase treatment, which caused dephosphorylation of Mover at T13, but should also lead to general dephosphorylation of SV proteins. This raises two possibilities for the mechanisms by which Mover is attached to SVs: 1) phosphorylation of Mover itself- at T13 or any of the other predicted phosphorylation sites- may be necessary for its association with SVs, or 2) phosphorylation of interacting partners of Mover may be necessary for its association with SVs. Although we cannot distinguish between these two possibilities at this point, the fact that SV bound Mover is phosphorylated at T13 is consistent with the former scenario. Future experiments using viral expression of a T13 phospho-deficient mutant in a Mover knockout background would allow a direct test of this notion.

Working in a knockout background will be essential for future experiments because our yeast 2-hybrid, co-immunoprecipitation and Vero cell data reveal a strong tendency of Mover to form homomers. In a wild-type background loss of function mutants lacking SV targeting signals may piggyback on endogenous Mover and thereby be targeted to SVs. We have identified a region between amino acids 52 and 91 of Mover that is required both for homomeric interaction in Vero cells and for targeting to SVs in neurons. This may indicate that homomeric interaction – which could create a targeting competent conformation – is required for presynaptic targeting of Mover. Such a scenario has been described for synapsins, which requires heterodimerization for presynaptic targeting [38]. Alternatively, this region may only mediate homomerization and thereby allow piggybacking of SV-targeting deficient mutants onto endogenous Mover. In this scenario, a presynaptic targeting signal might reside outside this region, between amino acids 1 and 52. In either case, our data show that a 39-amino acid region is required for Mover homomerization, and the same region or a different region within the first 90 amino acids of Mover is required for presynaptic targeting. Clearly, a knockout background is necessary to distinguish between these possibilities.

A striking result of our study is that deletion of almost any region of Mover causes a defect in homomeric interaction, i.e. regions of both the N and C termini are required. It will be important to test whether these regions undergo intramolecular interactions that allow for proper folding of Mover and its subsequent ability to form homomers. Alternatively, each of these regions may contribute to intermolecular interactions between Mover molecules.

The function of Mover remains to be elucidated. However our data clearly point to a presynaptic role of Mover on SVs, given its localization, and implicate phosphorylation and homomeric interaction in its function. Our data suggest it is likely that Mover remains phosphorylated and attached to SVs during synaptic activity. Dephosphorylation of Mover – if it occurs in vivo – might instead be important for Mover localization during development or synaptic plasticity. In particular, in early development Mover may have roles unrelated to synaptic transmission, given that Mover is under the control of the transcription factor P73, which is involved in brain development [16].

In addition, through interacting with bassoon, Mover may provide a link between SVs and the active zone. Interestingly, the C-terminal region of bassoon, which binds to Mover in a yeast-2-hybrid assay [12], is located within 50 nm of the active zone [39] raising the possibility that Mover provides a bridge between bassoon and docked vesicles. It will be interesting to find out how such vertebrate-specific molecular interactions contribute to synaptic assembly and function.
